# *FGFR4* c.1162G > A (p.Gly388Arg) Polymorphism Analysis in Turkish Patients with Retinoblastoma

**DOI:** 10.1155/2020/9401038

**Published:** 2020-12-30

**Authors:** Demet Akdeniz Odemis, Seref Bugra Tuncer, Arash Adamnejad Ghafour, Khariga Jabbarli, Yasemin Gider, Betul Celik, Gozde Kuru Turkcan, Ozge Sukruoglu Erdogan, Seda Kilic Erciyas, Mukaddes Avsar, Rejin Kebudi, Sema Buyukkapu Bay, Samuray Tuncer, Hulya Yazici

**Affiliations:** ^1^Istanbul University, Oncology Institute, Department of Basic Oncology, Division of Cancer Genetics, Istanbul, Turkey; ^2^Istanbul University, Oncology Institute, Division of Pediatric Hematology-Oncology, Istanbul, Turkey; ^3^Istanbul University, Istanbul Medical Faculty, Department of Ophthalmology, Istanbul, Turkey

## Abstract

**Purpose:**

Various molecular variations are known to result in different gene variants in the *FGFR4* gene, known for its oncogenic transformation activity. The goal of this study was to investigate the *FGFR4* p.Gly388Arg variant that plays role in the progression of cancer and retinal growth and may be an effective candidate variant in the Turkish population in retinoblastoma patients with no *RB1* gene mutation.

**Methods:**

Using the Sanger sequencing methods, the *FGFR4* p.Gly388Arg variant was bidirectionally sequenced in 49 patients with non-*RB1* gene mutation in retinoblastoma patients and 13 healthy first-degree relatives and 146 individuals matched by sex and age in the control group.

**Results:**

In Turkish population-specific study, the *FGFR4* p.Gly388Arg variant was found in 27 (55.1 percent) of 49 patients; mutation was found in 7 (53.8 percent) of these patients' 13 healthy relatives screened. When *FGFR4* p.Gly388Arg mutation status is evaluated in terms of 146 healthy controls, in 70 (47.9 percent) individuals, mutation was observed. Our analysis showed that the *FGFR4* p.Gly388Arg allele frequency, which according to different databases is seen as 30 percent in the general population, is 50 percent common in the Turkish population.

**Conclusions:**

In patients with advanced retinoblastoma who were diagnosed with retinoblastoma prior to 24 months, the *FGFR4* p.Gly388Arg allele was found to be significantly higher. As a result, these results indicate that the polymorphism of *FGFR4* p.Gly388Arg may play a role in both the development of tumors and the progression of aggressive tumors.

## 1. Introduction

Retinoblastoma is the most common primary intraocular malignancy in childhood and originates from primitive stem cells in the retina's nuclear layer [1]. Important genetic factors have been shown to play a role in the development of retinoblastoma [2]. According to existing literature, mutations in the retinoblastoma gene (*RB1* (RefSeq NM-000321.2 and chromosome 13 coordinates in hg19)) [3] are believed to trigger the disease. However, how the disease progresses in patients who do not have an *RB1* gene mutation scanned using the MLPA methods to assess minor insertions and deletions and sequencing of Sanger and large rearrangements is not clear. Many molecular changes that play an important role in the pathogenesis of tumors and the results of these changes have been seen in cancer patients in recent years. Growth factors, the oncogenic function of which was first identified, were found to play a role in the formation and development of tumors, activation of the cell cycle, formation of blood vessels, and escape from apoptotic control [4].

Today, the first isolated and most studied members of the family of fibroblast growth factor (FGF) are FGF-1 and FGF-2, which are considered to consist of at least 23 structurally related polypeptic mitogens. In several pathophysiological processes, such as embryonic development, differentiation, neuronal survival, wound repair, and tumor formation, FGF family members play important roles in a wide range of tissues and cell types [5, 6]. However, functions in angiogenesis, differentiation, and survival have been found in the FGF family [7]. With 5 distinct but very similar high affinity cell surface tyrosine kinase receptors (FGFR1, FGFR2, FGFR3, *FGFR4*, and FGFR5) [8], the FGF family has demonstrated its biological activities [8].

A member of the family of fibroblast growth factor (FGF) genes, the *FGFR4* gene is also noted for its oncogenic transformation activity. Various molecular variations in the *FGFR4* gene have been shown to lead to different variants of the gene. The *FGFR4* variant (GRCh37 Chr5: 176520243, NM 002011.4: c.1162G > A p.Gly388Arg, rs351855 at the genotype level) is one of these variants, triggering the conversion of glycine amino acid to arginine amino acid at codon 388 [9]. *FGFR4* p.Gly388Arg variant has been found to be correlated with development and disease prognosis of breast cancer [10], cervical cancer [11], hepatocellular carcinoma [12], thyroid cancer [13], gastric cancer [14], soft tissue tumors [15], lung cancer [9], colon cancer [16], prostate cancer [17], and head and neck tumors [18].

The *FGFR4* gene is known to exert neuroprotective effects against the degeneration of retinal photoreceptors. The *FGFR4* gene acts on the FGF-19 ligand, a molecule essential for photoreceptor ocular tissue formation and expressed by the embryonic retina. In mediating tumor growth and development, overexpression of *FGFR4* and amplification of its target receptor FGF19 play a significant role. The abnormal signaling of FGF19-*FGFR4* has been shown to influence the downstream signaling cascade involving particular tumorigenic events including cancer cell proliferation, resistance to apoptosis, and metastasis [19]. In this context, it can be thought that the variant of *FGFR4* p.Gly388Arg in the *FGFR4* gene, which is known to play a role in cancer progression and retinal development, may be a candidate mechanism and marker gene that triggers retinoblastoma formation. The effect of the *FGFR4* p.Gly388Arg variant on retinoblastoma pathogenesis has been investigated in this planned study. This research indicates that the variant of *FGFR4* p.Gly388Arg may have a population-specific polymorphism as a result of analysis performed in retinoblastoma patients and control groups after sequencing by the Sanger sequencing process.

## 2. Materials and Methods

### 2.1. Study Design and Patient Characteristics

The study was approved by the Local and Clinical Research Ethics Committee of Istanbul University (number of ethical approval: 2016-360), according to the tenets of the Declaration of Helsinki (JAMA 1997; 277 : 925–926). Written informed consent was obtained from all participants or parents of children under 18 years of age.

All patients consist of patients who applied to Istanbul University, Oncology Institute, Division of Pediatric Hematology-Oncology Istanbul University, Istanbul Medical Faculty, Department of Ophthalmology between 2011 and 2018. Although the study group was composed of a total of 208 individuals, all minor deletions and insertions were scanned predominantly by Sanger sequencing, large rearrangements were scanned with MLPA, and 49 individuals were selected for diagnosis of non-*RB1* gene mutation retinoblastoma. The mutation status of 13 healthy first-degree relatives of the patients was also assessed in order to gain information on the family inheritance of the *FGFR4* p.Gly388Arg variant. Differences between 146 age- and sex-matched healthy controls and patients with retinoblastoma were examined in order to determine the effect of this variant on retinoblastoma pathogenesis.

### 2.2. Features of the FGFR4 p.Gly388Arg Variant

In the NCBI reference sequence database (RefSeq) [20] and in algorithms such as Polymorphism Phenotyping (PolyPhen) [21] and SIFT [22], the *FGFR4* p.Gly388Arg variant has been scanned. Data from the Catalog of Somatic Mutations in Cancer (COSMIC) [23], the ClinVar database [24], and the Online Mendelian Inheritance in Man (OMIM) catalogue [25] were used to test the relationship between the variant and the disease. The dbSNP [26], the Exome Aggregation Consortium (ExAC), the Genome Aggregation Database (gnomAD) [27], and the Ensemble 1000 Genome Project [28] were analyzed for information on the frequency and occurrence of variants in the population. The information gathered from the *FGFR4* p.Gly388Arg variant data banks is shown in Table 1.

### 2.3. DNA Samples and Quality Checks

Peripheral blood samples were taken from all individuals involved in the analysis, and the Ficol (Sigma-Aldrich, Darmstadt, Germany) separation method was used for lymphocyte isolation. In compliance with the manufacturer's instructions, DNA isolation from these lymphocytes was carried out using the QIAamp DNA mini kit (Qiagen, 40724 Hilden, Germany). All DNA sample quality controls were carried out using the Qubit fluorimeter (ThermoFisher Science, Paisley PA4 9RF, UK) unit.

### 2.4. PCR Reaction and DNA Sequencing

For the sequencing of the *FGFR4* p.Gly388Arg variant located in the 9^th^ Exon of the *FGFR4* gene, two separate primers were designed in our study: forward (5′-TGACCAGTTTGTCTGTCTGTG-3 ′) and reverse (5′-AGAACTGCAAAGTGGGAGAC-3′). Patient and control group samples were amplified by polymerase chain reaction using the designed primer pair (PCR). The PCR process was performed with a 50 *μ*L volume of 5 *μ*l Taq polymerase enzyme and a genomic DNA of 150 ng. Using the ABI Prism 3730 DNA Analyzer (Applied Biosystems, Foster City, CA) system, the PCR products obtained were bidirectionally sequenced and sequencing data were analyzed using SnapGene Viewer 4.0.5 Software.

### 2.5. Statistical Analysis

Our main objective was to obtain information on the family inheritance of the variant *FGFR4* p.Gly388Arg and to assess its effect on the pathogenesis of retinoblastoma. In order to estimate the distribution of the *FGFR4* p.Gly388Arg variant in the population and to compare the genotype variations between the patient and control groups, a chi-square (*X*^2^) test was carried out using categorical variables. In addition, to equate the clinicopathological and epidemiological parameters with the *FGFR4* variant p.Gly388Arg, the *X*^2^ test was used. A *p* value of <0.05 was found statistically relevant. All statistical analyses were performed using Windows (SPSS, Chicago, IL, USA) version 21.0 SPSS software.

## 3. Results

Scope of work, a total of 8 criteria, including stratification analysis, were evaluated in terms of clinicopathological characteristics: month of diagnosis, sex, diagnosis, leukocoria, strabismus, glaucoma, and treatment.  Table 2 displays all the clinicopathological characteristics in the cases. The diagnostic age (months) of the patients ranged from 1 to 216 months, while the mean age was 21 months. Although 31 (63.3 percent) of 49 patients with retinoblastoma were diagnosed prior to 24 months, 18 (36.7 percent) were diagnosed after 24 months. 16 (59.3%) of patients diagnosed before 2 years of age and 11 (40.7%) of patients diagnosed after 2 years of age were positive for the p.Gly388Arg *FGFR4* mutation (*p*=0.04^*∗*^). In terms of the *FGFR4* p.Gly388Arg mutation, 18 (51.4 percent) of 35 (56.5 percent) female patients and 16 (59.3 percent) of 27 (43.5 percent) male patients were considered positive. In 25 (58.1%) of 43 (87.7%) patients with unilateral retinoblastoma, the *FGFR4* p.Gly388Arg mutation was detected; in 2 (33.3%) of 6 (12.3%) patients with bilateral retinoblastoma, the *FGFR4* p.Gly388Arg mutation was identified. In 25 (58.1%) of 43 (87.7%) patients with unilateral retinoblastoma, the *FGFR4* p.Gly388Arg mutation was detected; in 2 (33.3%) of 6 (12.3%) patients with bilateral retinoblastoma, the *FGFR4* p.Gly388Arg mutation was identified. 29 (59.2%) patients with strabismus disease applied to the doctor, 19 (65.5%) of these patients had esodeviation, and 10 (34.5 percent) had exodeviation. In 15 (51.7 percent) of these strabismus patients, the *FGFR4* p.Gly388Arg mutation was found to be positive. In 7 (14.3 percent) of the patients at the time of diagnosis, glaucoma was seen, but only 2 (28.6 percent) of these patients had mutations.

Although 38 (77.6%) of 49 patients with retinoblastoma were diagnosed with advanced stage (group C, group D, or group E) retinoblastoma, 11 (22.4%) were diagnosed with early-stage retinoblastoma, when the clinical stages of the patients were assessed (group A and group B). Although 24 (88.8%) of the 27 *FGFR4* p.Gly388Arg mutation patients were in the advanced stage, 3 (11.1%) were diagnosed early. A statistically relevant association between early stage and advanced stage patients (*p*=0.031) was found in terms of *FGFR4* p.Gly388Arg mutation when the clinical stage and *FGFR4* p.Gly388Arg mutation status were evaluated. 26 (53.1 percent) had intra-arterial chemotherapy (IAC), 19 (38.8 percent) had systemic chemotherapy (CT) for chemoreduction, 2 (4.1 percent) had regional radiotherapy (RT), 7 (14.3 percent) had local ophthalmic treatment (LOT) (cryotherapy, thermotherapy, and laser therapy), and 20 (40.8 percent) were determined to have undergone treatment (LOT) (cryotherapy, thermotherapy, and laser therapy) and 20 (40.8 percent) enucleation of eye. There was no statistically relevant association between the p.Gly388Arg mutation in *FGFR4* and the patients' treatment choices (*p* ≥ 0.05).

It was found that 3 (6.1 percent) patients had a history of first-degree-associated retinoblastoma when evaluating the association of the *FGFR4* p.Gly388Arg variant with family history. These were all patients with heterozygous GA alleles; c. [1162G > G]; the [1162G > A] mutation was introduced. In this study, in patients with a family history of retinoblastoma, the *FGFR4* p.Gly388Arg mutation rate was recognized as “almost significant” (e.g., *p*=0.08^*∗∗*^). 36 (73.5 percent) patients have been identified when the presence of tumors other than retinoblastoma in the patient family is investigated. Of these patients, 20 (55.5 percent) were found to be positive for the p.Gly388Arg *FGFR4* mutation and 16 (45.5 percent) were negative (*p*=0.94). It was reported that the family members of 31 (63.3%) patients were included in each of these occupational groups when the next three generations are studied in terms of risk evaluation of occupational groups (machinists, welders, metal industry, textile, building, and military). In 19 (61.3%) of these patients, the *FGFR4* p.Gly388Arg variant was identified (*p*=0.07 is nearly significant).  Table 3 indicates the association of the *FGFR4* p.Gly388Arg variant with family history.

**Figure 1:**
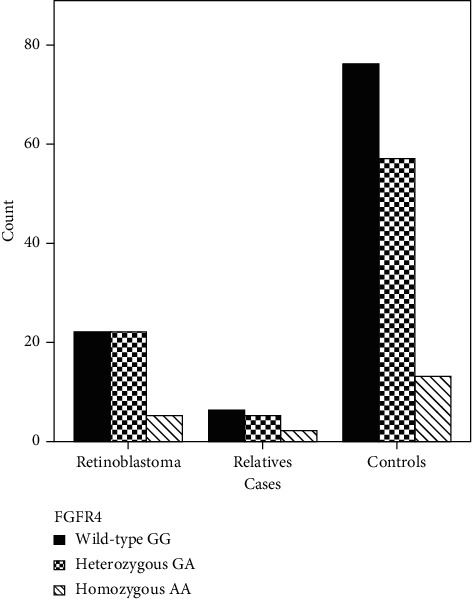
The distribution of FGFR4 p.Gly388Arg (c.1162G > A) variant in retinoblastoma patients, relatives, and control group.

The Wild-type GG allele rate was 104 (50%), heterozygous GA allele rate 84 (40.4%), and homozygous AA allele rate 20 (9.6%) among 208 samples. In 27 (55.1 percent) of 49 patients, the *FGFR4* p.Gly388Arg variant was found positive; 22 (81.5 percent) carried the heterozygous GA allele and 5 (18.5 percent) carried the homozygous AA allele. 5 (71.4 percent) of the 7 individuals were found to have the heterozygous GA allele and 2 (28.6 percent) the homozygous AA allele as a result of the screening of 13 healthy relatives of these patients. When the mutation status of *FGFR4* p.Gly388Arg is tested in terms of 146 healthy controls, mutations were observed in 70 individuals, 57 (81.4%) were heterozygous GA allele, and 13 (18.6%) were homozygous AA allele. In terms of the *FGFR4* p.Gly388Arg mutation (*p*=0.86), there was no statistically significant difference between the patient and control groups.  Table 4 and Figure 1 display the distribution of the *FGFR4* p.Gly388Arg (c.1162G > A) variant in patients, relatives, and control groups.

## 4. Discussion

Cancer is a hereditary disorder that occurs in several genes as a result of mutations that play role in the division, proliferation, and death of cells. Retinoblastoma develops in the eye and is the most prevalent childhood intraocular malignancy. Important genetic factors are believed to play a role in the development of retinoblastoma. It is understood, according to current literature, that the disease is caused by mutations in a gene called the retinoblastoma. It is not clear, however, how the disorder progresses in patients who are found to be negative as a result of *RB1* gene mutation scans.

The *FGFR4* gene is known to exert neuroprotective effects against the degeneration of retinal photoreceptors. In photoreceptors, *FGFR4* expression affects a particular ligand, FGF-19. FGF-19 is a molecule that is essential and expressed by the embryonic retina for the development of ocular tissue. There are also several studies in the literature that examine the possible role of FGF-19 in the mechanism of photoreceptors. Siffroi-Fernandez et al., in 2008, stressed the neuroprotective effects of FGF-19 on mammalian photoreceptors [29]. Lang et al., in 2018, showed that overexpression of *FGFR4* and amplification of its target FGF19 receptor played an important role in mediating tumor growth and progression [19]. Villalonga et al, in their 2018 report, emphasized that the variant *FGFR4* p.Gly388Arg initiated tumor progression by stimulating the expression of N-cadherin protein in lung cancer patients [9]. The variant *FGFR4* p.Gly388Arg has been shown to penetrate the STAT3 molecule into the cell membrane and impact cell surface molecules, thereby accelerating the progression of cancer and becoming an important risk factor for the progression of the disease [30]. Bange et al. observed in their study that the *FGFR4* p.Gly388Arg variant was associated with cancer progression and cell motility [31]. A candidate mechanism and marker gene that activates the formation of retinoblastoma in this context may be considered to be the *FGFR4* p.Gly388Arg variant in the *FGFR4* gene, which is known to be involved in the development of cancer and retinal growth.

In this study, the effect of the *FGFR4* p.Gly388Arg variant on 49 nonmutated patients with retinoblastoma of the *RB1* gene, their 13 healthy relatives and 146 age- and gender-matched control groups, and the *FGFR4* p Gly388Arg variant on patients with retinoblastoma and control groups used the bidirectional sequencing Sanger Sequencing process. As a result, we have investigated whether there could be a mechanism that initiates retinoblastoma oncogenesis.

Our first goal in this study was to establish if the variant *FGFR4* p.Gly388Arg may be another primary mechanism in the formation of retinoblastoma other than the *RB1* gene. In the research, no statistically significant difference in mutation in the *FGFR4* p.Gly388Arg variant was identified between the patient and control groups. It was also assumed that the *FGFR4* p.Gly388Arg variant was a population-specific polymorphism.

The most significant finding of this study is the explanation of the effect of a polymorphism in patients who have no *RB1* mutation but are diagnosed with RB, triggering the conversion of the glycine amino acid to the amino acid of arginine at the 388 codon of the human *FGFR4* gene. When the *FGFR4* p.Gly388Arg variant minor allele frequency is screened in the ExAC and Genome Aggregation Database (gnomAD) datasets, it appears to be a known polymorphism in the general population with a MAF ratio of 0.3209. In our Turkish population-specific research, however, the *FGFR4* p.Gly388Arg variant was found to be positive in 27 (55.1 percent) of 49 patients; mutation was found in 7 (53.8 percent) of these patients' 13 healthy relatives screened. When *FGFR4* p.Gly388Arg mutation status is evaluated in terms of 146 healthy controls, in 70 (47.9 percent) individuals, mutation was observed. As a result, in 104 (50 percent) of 208 individuals screened in total, the *FGFR4* p.Gly388Arg mutation was detected. In view of all these findings, our analysis has shown that the frequency of *FGFR4* p.Gly388Arg allele, which according to various databases is seen as 30 percent in the general population, is prevalent in the Turkish population at a rate of 50 percent.

Nonetheless, our statistical study clearly showed that, in advanced stage patients diagnosed with retinoblastoma prior to 24 months, the *FGFR4* p.Gly388Arg allele was significantly higher. As a result, these findings suggest that the *FGFR4* p.Gly388Arg allele may play a role in both aggressive tumor progression and tumor formation. Furthermore, taking into account the status of the *FGFR4* p.Gly388Arg allele, this polymporphism can be considered to be a novel alternative for predicting clinical development and assessing the stage of disease in patients with advanced stage retinoblastoma. Studies on a wider community of patients with longer follow-up data will help to test this theory. The findings to be obtained from future studies are expected to contribute to the literature on the pathogenesis, etiology, and genetic origin of retinoblastoma and to direct the next generation in terms of early detection and options for treatment. In addition, genetic testing in the prenatal and preimplantation phases in new family pregnancies may be conducted to predict the disease until the baby is born, or the gene-borne disease in the family can be fully removed by in vitro fertilization (ivf).

## Figures and Tables

**Table 1:** Characteristics of *FGFR4* p.Gly388Arg variant.

Genes	Exon	Variants	Rs number	Type of variants	Primary region of effected in COSMIC	Cited cancer in COSMIC	MAF	SIFT^a^	PolyPhen^b^	ClinVar
*FGFR4* (NM_002011.4)	Exon 9	c.1162G > A (p.Gly388Arg)	rs351855	missense_variant	Thyroid, soft tissue, soft tissue	Others, rhabdomyosarcoma, hemangioblastoma	0.3209	Tolerated (0.2)	Possibly damaging (0.742)	Pathogenic

^a^SIFT value predication ranges from 0 to 1. Prediction of damaging or tolerated if the score shows ≤0.05 or >0.05, respectively. ^b^Polyphen value predication ranges from 0 to 1. A variant is appraised qualitatively, as benign (0.00–0.15), possibly damaging (0.16–0.85), or probably damaging (0.86–1.00). COSMIC, Catalogue of Somatic Mutations in Cancer; MAF; minor allele frequency from the ExAC and gnomAD datasets; na, not available.

**Table 2:** Association of *FGFR4* p.Gly388Arg variant with clinicopathologic variants.

Clinicopathologic features	Wild-type GG no. (%)	Heterozygous GA no. (%)	Homozygous AA no. (%)	Total no. (%)	Significance, *P* value
Age (months), mean 21 ± 4, range (1–216)					
<24 months	15 (68.2%)	11 (50%)	5 (100%)	31 (63.3%)	0.04 ^*∗*^
≥24 months	7 (31.8%)	11 (50%)	0 (0%)	18 (36.7%)	
Total	22	22	5	49	

Sex					
Female	15 (68.2%)	12 (54.5%)	1 (20%)	28 (57.1%)	0.137
Male	7 (31.8%)	10 (45.5%)	4 (80%)	21 (42.9%)	
Total	22	22	5	49	

Diagnosis					
Unilateral	18 (81.8%)	20 (90.9%)	5 (100%)	43 (87.8%)	0.444
Bilateral	4 (18.2%)	2 (9.1%)	0 (0%)	6 (12.2%)	
Total	22	22	5	49	

Leukocoria					
Yes	17 (77.3%)	16 (72.7%)	4 (80%)	37 (75.5%)	0.912
No	5 (22.7%)	6 (27.3%)	1 (20%)	12 (24.5%)	
Total	22	22	5	49	

Strabismus					
Esodeviation	10 (45.5%)	7 (31.8%)	2 (40%)	19 (38.8%)	0.929
Exodeviation	4 (18.1%)	5 (22.7%)	1 (20%)	10 (20.4%)	
No	8 (36.4%)	10 (45.5%)	2 (40%)	20 (40.8%)	
Total	22	22	5	49	

Glaucoma					
Yes	5 (22.7%)	2 (9.1%)	0 (0%)	7 (14.3%)	0.201
No	17 (77.3%)	20 (90.9%)	5 (100%)	42 (85.7%)	
Total	22	22	5	49	

Stage					
Early stage	8 (36.4%)	3 (13.6%)	0 (0%)	11 (22.4%)	0.031 ^*∗*^
Late stage	14 (63.6%)	19 (86.4%)	5 (100%)	38 (77.6%)	
Total	22	22	5	49	

Treatment					
IAC					
Yes	10 (45.5%)	12 (54.5%)	4 (80%)	26 (53.1%)	0.370
No	12 (54.5%)	10 (45.5%)	1 (20%)	23 (46.9%)	
Total	22	22	5	49	

CT					
Yes	10 (45.5%)	8 (36.4%)	1 (20%)	19 (38.8%)	0.546
No	12 (54.5%)	14 (63.6%)	4 (80%)	30 (61.2%)	
Total	22	22	5	49	

RT					
Yes	2 (9.1%)	0 (0%)	0 (0%)	2 (4.1%)	0.278
No	20 (90.9%)	22 (100%)	5 (100%)	47 (95.9%)	
Total	22	22	5	49	

Surgery (enucleation)					
Yes	11 (50%)	9 (40.9%)	0 (0%)	20 (40.8%)	0.05
No	11 (50%)	13 (59.1%)	5 (100%)	29 (59.2%)	
Total	22	22	5	49	

LOT					
Yes	3 (13.6%)	4 (18.2%)	0 (0%)	7 (14.3%)	0.573
No	19 (86.4%)	18 (81.8%)	5 (100%)	42 (85.7%)	
Total	22	22	5	49	

Wild-type GG allele; c. [1162G > G]; [1162G > G], heterozygous GA allele; c. [1162G > G]; [1162G > A], homozygous AA; c. [1162G > G]; [1162A > A]. Early stage, group A, group B, and group C; late stage, group D or group E; IAC, intra-arterial chemotherapy; CT, chemotherapy; RT, radiotherapy; surgery, enucleation of eye; LOT, local ophthalmic treatment (cryotherapy, thermotherapy, and laser therapy);  ^*∗*^*p* < 0.05 is significant.

**Table 3:** Association of *FGFR4* p.Gly388Arg variant with family history.

Family history	Wild-type GG no. (%)	Heterozygous GA no. (%)	Homozygous AA no. (%)	Total no. (%)	Significance, *p* value
RB in family					
Yes	0 (0%)	3 (13.6%)	0 (0%)	3 (6.1%)	0.08
No	22 (100%)	19 (86.4%)	5 (100%)	46 (93.9%)	
Total	22	22	5	49	

OT in family					
Yes	16 (72.7%)	16 (72.7%)	4 (80%)	36 (73.5%)	0.94
No	6 (27.3%)	6 (27.3%)	1 (20%)	13 (26.5%)	
Total	22	22	5	49	

RAOG in family					
Yes	12 (54.5%)	14 (63.6%)	5 (100%)	31 (63.3%)	0.07
No	10 (45.5%)	8 (36.4%)	0 (0%)	18 (36.7%)	
Total	22	22	5	49	

Wild-type GG allele; c.[1162G > G]; [1162G > G], heterozygous GA allele; c. [1162G > G]; [1162G > A], homozygous AA; c. [1162G > G]; [1162A > A]. RB, retinoblastoma; OT, other tumors (brain tumor, breast cancer, lung cancer, thyroid cancer, and prostate cancer); RAOG, risk assessment of occupational groups.

**Table 4:** Distribution of *FGFR4* p.Gly388Arg (c.1162G > A) variant in patients, relatives, and control group.

*FGFR4* p.Gly388Arg (c.1162G > A)	Patients no. (%)	Relatives no. (%)	Control no. (%)	Total no. (%)	Significance, *p* value
Wild-type GG	22 (21.2%)	6 (5.7%)	76 (73.1%)	104 (50%)	0.86
Heterozygous GA	22 (26.2%)	5 (6%)	57 (67.8%)	84 (40.4%)	
Homozygous AA	5 (25%)	2 (10%)	13 (65%)	20 (9.6%)	
Total	49	13	146	208	

Wild-type GG allele; c. [1162G > G]; [1162G > G], heterozygous GA allele; c. [1162G > G]; [1162G > A], homozygous AA; c. [1162G > G]; [1162A > A].

## Data Availability

The data used to support the findings of this study are restricted by the Local and Clinical Research Ethics Committee of Istanbul University in order to protect patient privacy.
